# SCRUM: A simple model for estimating uptake of nitrogen and water by field crops

**DOI:** 10.1016/j.mex.2026.103981

**Published:** 2026-06-02

**Authors:** Edith N. Khaembah, Rogerio Cichota, Megan Gee, Hamish E. Brown

**Affiliations:** New Zealand Institute for Bioeconomy Science Limited, Private Bag 4704, Christchurch Mail Centre, Christchurch 8140, New Zealand

**Keywords:** Crop model, Simple, Nitrogen balance, Water balance, Farm systems modelling

## Abstract

This paper describes the **S**imple **C**rop Resource **U**ptake **M**odel (SCRUM), a configurable generic crop model implemented in APSIM Next Generation’s Plant Modelling Framework. SCRUM simulates crop development using a nine‑phase phenology coupled to a simplified plant module consisting of stover, product, root, and nodule (only in legumes) and a thermal‑time‑driven trajectory for daily potential growth. Water limitation is computed through APSIM’s canopy energy balance (MicroClimate). Nitrogen uptake is determined by organ growth and tissue nitrogen targets, constrained by soil mineral nitrogen supply and supplemented by biological fixation when enabled. The generic and simple nature of SCRUM makes it easy for new crops to be configured using expert knowledge, enabling rapid set‑up for species and end‑uses where comprehensive crop models are unavailable. Model evaluation using field data indicated that SCRUM, in the APSIM framework, is a promising a tool that can utilised in the analysis of nitrogen and water management practices of across a range of conditions.

• A generic, configurable crop model for rapid parameterization of data‑limited species and end‑uses.
• Coupled water stress (canopy energy balance) and nitrogen dynamics (demand targets, supply constraints, optional fixation).
• A transparent model description including structure, parameter inputs and assumptions.

## Graphical abstract

**Figure:**
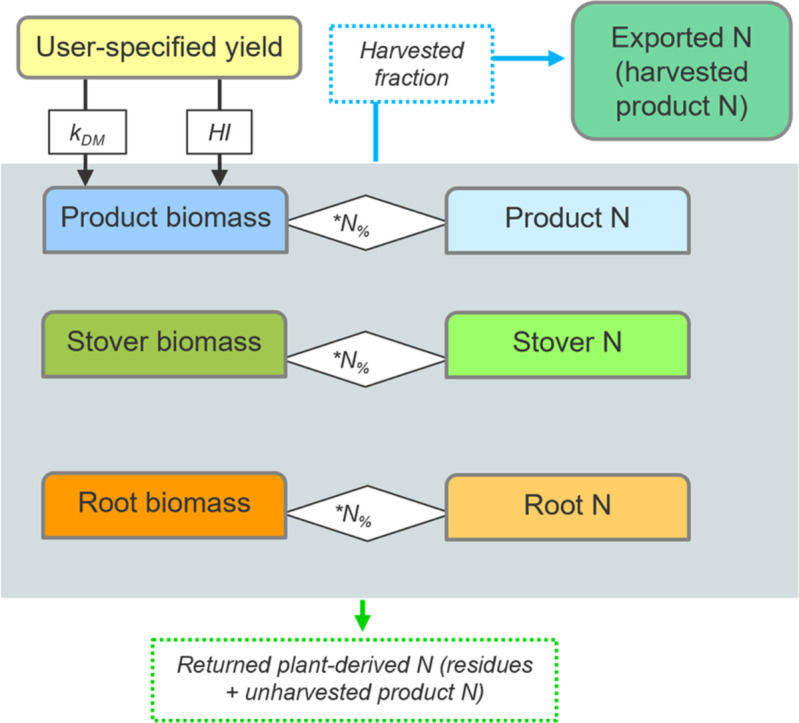

**Specifications table**

**Table:** 

**Subject area**	Agricultural and Biological Sciences
**More specific subject area**	Crop modelling
**Name of your method**	Simple Crop Resource Uptake Model (SCRUM)
**Name and reference of original method**	None
**Resource availability**	SCRUM validation

## Background

Systems models are widely used for assessing the environmental impacts of crops and the risks of nitrogen and water stress under different scenarios. Crop models, in particular, are powerful tools that enable faster, risk-free, resource-efficient and scalable analysis of crop response to management practices and environmental factors [[1], [2], [3]]. These models have an important role in farm management decision-making, especially as agriculture faces the dual challenge of boosting food production while safeguarding natural resources under growing climate threats [4]. The application of crop modelling in agricultural decision making is often limited by scarcity or non-existence of good quality data and availability of experts for developing new crop models [5,6]. One approach to overcoming this limitation is to create a generic model based on processes common to crop species. This approach has been previously adopted in purpose-built crop model development projects [7,8]. The development of a generic model can include the capability to input parameter files that specify the unique characteristics of individual crops and cultivars. This flexibility is particularly beneficial for crop rotations, which are complex systems involving multiple crops and may include new or less researched crops.

The development of new models can now benefit from software infrastructures that support design with varying degrees of complexity. One such platform is the **A**gricultural **P**roduction **S**ystems s**IM**ulator (APSIM; www.apsim.info), particularly its Plant Modelling Framework [9], which enables flexible and modular model development. APSIM is a well-established and widely used tool in research to support resource management, assess land use suitability, and forecast agricultural production under climate change scenarios [[10], [11], [12], [13]].

This paper presents the Simple Resource Uptake Model (SCRUM), designed to estimate water and nitrogen uptake in annual cropping systems. The model structure is based on common plant processes and incorporates user-defined input variables, allowing customization to reflect species- or cultivar-specific characteristics without altering the core framework.

## Method details

### SCRUM overview

SCRUM was developed using the APSIM Plant Modelling Framework [9] to estimate water and nitrogen uptake in field crops. It is particularly useful for evaluating water and nitrogen balances, as well as stress responses under limited resource conditions, in systems with crops for which fully mechanistic plant models are not available or where simplified representations of those crops are sufficient. SCRUM features a generic structure comprising of a phenology component and a plant module. Its integration within APSIM enables daily simulation of potential crop growth, stress responses and dynamic interactions with other system components, including soil water, soil nitrogen, climate and crop management modules [14].

### SCRUM plant module

The plant model consists of four organs: stover, product, root and nodule. Stover is a simple leaf organ and therefore, responsible for simulating crop canopy and biomass assimilation. The biomass in the stover organ represents unharvested portions of the plant. Its composition depends on the crop species, the intended end-use, or a combination of both. For instance, in oats grown for grain, stover includes the entire leaf and stem biomass, essentially the above-ground biomass excluding the grain. In contrast, when oats are cultivated for forage (green feed), the stover component refers to the residual leaf and stem biomass remaining after harvest.

The SCRUM product is a generic organ class representing plant parts that are harvested and removed from the field. Like stover, its composition depends on the crop species, its intended end-use, or both. Product forms vary widely across crops. For example, grain in wheat and oats grown for grain; above-ground biomass in leafy vegetables and forage crops like green feed oats; tubers in potatoes; taproots in carrots and beetroots; fruit in tomatoes; and bulbs in onions.

The SCRUM root is a below-ground organ that extracts water and nitrogen from the soil to support plant growth. It also determines how roots are distributed throughout the soil profile. At harvest, the root biomass is returned to the soil as fresh organic matter.

The SCRUM nodule is an organ responsible for biological nitrogen fixation. In the model, a switch is included to enable presence of this organ only when leguminous crops are simulated.

### SCRUM phenology

The phenology model consists of nine sequential phases, each categorized by distinct starting and ending developmental stages (Table 1). Progression through each phenological phase is governed by thermal time (measured in degree-days), calculated using cardinal temperatures (minimum, optimum and maximum), consistent with other crop models implemented in APSIM [15,16].

**Table 1:** Details of the phenological stages in the Simple Crop Resource Uptake Model (SCRUM; based on the Agricultural Production Systems sIMulator (APSIM)). ProportionTT denotes the fraction of total thermal time (TT) time allocated to the start of a phase, while ProportionMaxDM is the maximum proportion of the crop’s potential dry matter accumulated at the end of each phase.

**Phase number**	**Phase name**	**Phase start stage**	**Phase end stage**	**ProportionTT**	**ProportionMaxDM**
1*	Seed	Seed	Emergence	−0.0517	0.004
2	Seedling	Emergence	Seedling	0.0001	0.0067
3	Vegetative	Seedling	Vegetative	0.0501	0.011
4	Early reproductive	Vegetative	Early reproductive	0.5	0.5
5	Mid reproductive	Early reproductive	Mid reproductive	0.5847	0.7
6	Late reproductive	Mid reproductive	Late reproductive	0.6815	0.86
7	Maturity	Late reproductive	Maturity	0.7944	0.95
8	Ripe	Maturity	Ripe	0.9991	0.993
9*	Ready for harvest	Ripe	Unused	1.2957	0.999

⁎ Values <0 or >1 allow the model to position pre‑emergence and post‑maturity phases relative to the emergence–maturity timescale.

The model calculates thermal time from emergence to maturity (*TT_Emerge−__Mat_*) each time a crop is sown. This thermal time represents the accumulated heat units required for the crop to progress through its full cycle i.e. from emergence to physiological maturity. To determine this, the model uses one of the two user-provided crop cycle inputs:

1. Thermal time-based input, where the user specifies the total thermal time from establishment to harvest (*TT_Estab–Harvest_*). In SCRUM, ‘establishment’ refers to the user-defined starting developmental stage for the crop (e.g., seed or seedling), which may differ from sowing or emergence.
2. Calendar-based input, where the user provides specific establishment and harvest dates, and the model calculates thermal time using temperature data over that period.

Once the total thermal time is established, the model then applies fractionation factors to allocate portions of this thermal time to different phenological stages. For instance, the vegetative stage (stage 4) is completed when 50% of the total thermal time from crop emergence to maturity has been accumulated (ProportionTT = 0.5; Table 1). Similarly, the transition to the late reproductive stage (stage 7) occurs after ∼79% (ProportionTT = 0.7944) of the total thermal time has been accumulated.

To calculate TT_Emerge−__Mat_, the model uses the following equation when *TT_Estab–Harvest_* is provided:

(1) $$TT_{Emerge-Mat}=\frac{TT_{Estab-Harv\;}\;-\;TT_{Sow-Emerge}}{ProportionTT_{Estab-Harv}\;}$$

where:

• *TT_Sow–Emerge_* is a user-provided input
• *ProportionTT_Estab–Harv_* is ProportionTT (Table 1) for user-specified harvest stage minus the ProportionTT for the specified establishment phase.

If the user provides fixed establishment and harvest dates, the model first runs forward through the meteorological file to calculate TT_Estab–Harvest_ and then applies Eq. (1) to derive *TT_Emerge−__Mat_*.

SCRUM is designed to be configurable, enabling it to represent a wide range of crops and management practices. Crops can be established using different methods (e.g. seed or seedling) and harvested at various developmental stages, ranging from vegetative to ripe. These establishment and harvest stages, both defined by the user, determine the developmental phases a crop undergoes. The thermal time required to complete each phase is then calculated as:

(2) $$TT_{Phase_{i}}=\;TT_{Emerge-Mat}*\left(ProportionTT_{i+1}\;-\;ProportionTT_{i}\right)$$

where *i* is the phase number (see Table 1), TTEmerge*_–Mat_* is the thermal time from emergence to maturity as described in Eq. (1), and *ProportionTT* constants between the end and start of the *i^th^* phase provided in Table 1.

In addition to allocating thermal time across phenological stages, the model uses predefined thresholds (ProportionMaxDM listed in Table 1) to guide stage-specific dry matter distribution. These thresholds represent the proportion of the crop’s total potential dry matter that is expected to accumulate by the end of each stage. For instance, a maximum of 0.4% of the potential dry matter is allocated at emergence. This increases to 50% at the end of the early reproductive stage and reaches 95% at maturity. This staged allocation enables the model to simulate not only the timing of developmental transitions but also the progressive build-up of biomass, which is essential for estimating yield and resource uptake dynamics.

### Modelling plant growth

**Fig. 1:**
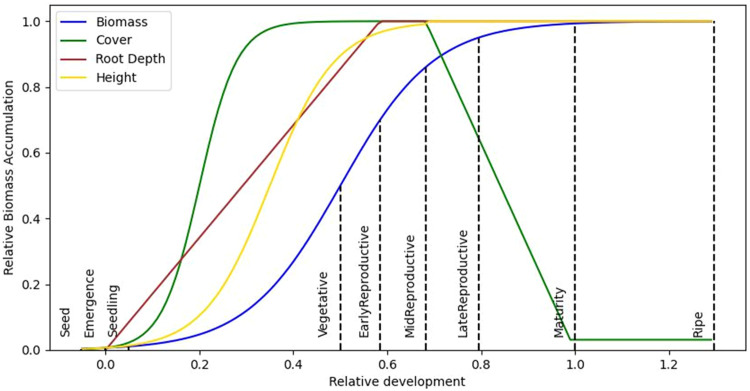
Relative biomass accumulation as a function of relative crop development as modelled by the Simple Crop Resource Uptake Model (SCRUM; based on the Agricultural Production Systems sIMulator (APSIM)), with additional curves showing crop cover, root depth and crop height. All variables are expressed as proportions of their respective maximum values.

Once phenological timing has been established, SCRUM uses it to regulate daily growth processes. Plant growth parameters including biomass accumulation, canopy cover, root depth and height are simulated daily using linear, sigmoidal or blended linear-sigmoidal functions. Fig. 1 illustrates how the temporal patterns of these parameters align with phenological stage transitions for a seed-sown crop harvested at the ripe stage. Biomass, crop height and green cover expansion follow a sigmoid growth pattern (Eq. (3)), while root depth and canopy senescence are modelled using linear functions:

(3) $$y=\;\frac{Value_{Max}}{1+exp^{\left(-\left(x-x_{o}\right)/b\right)}}$$

where:

• *Value_max_* represents the maximum potential value of the parameter (biomass, canopy cover, height)—the user specifies height and canopy cover directly, while biomass is derived from user-defined inputs of yield, harvest index and root proportion (see Table 2).

**Table 2:** Input parameters specified in a crop instance of the Simple Crop Resource Uptake Model (SCRUM; based on the Agricultural Production Systems sIMulator (APSIM)).

**Growth pattern and biomass partitioning parameters**	Units
Harvest index (0–1)	–
Product moisture content at harvest (0–1)	g/g
Root biomass proportion at maturity (0–1)	–
Root depth at maturity	mm
Crop height at maturity	mm
Maximum green cover (0–0.97)	–
Crop extinction coefficient (0–1)	–
Stage at which crop is typically harvested	–
**Crop N parameters**	
Nitrogen content at seedling stage (0–1)	g/g
Nitrogen content of product at harvest (0–1)	g/g
Nitrogen content of stover at harvest (0–1)	g/g
Nitrogen content of roots (0–1)	g/g
Potential nitrogen fixation by crop if legume (proportion; 0–1)	–
**Crop development as a function of temperature parameters**	
Crop base temperature	°C
Crop optimum temperature	°C
Crop maximum temperature	°C
Thermal-time (sowing to emergence)	°Cd
**Crop water requirement parameters**	
Maximum canopy conductance (0.001–0.016)	m/s
Net radiation at 50% of maximum conductance (50–200)	W/m^2^
• *x* is the accumulated thermal time (°Cd) from emergence.
• *x_o_* is the inflection point, marking the midpoint of the rapid growth phase.
• *b* is a shape parameter controlling the steepness of the curve.

For biomass, *x_o_* is calculated as 0.5*TT_Emerge−__Mat_ and *b* is calculated as 0.1*TT_Emerge−__Mat_. Daily biomass production is calculated as the differential of this function each day. The growth curve is scaled to have negligible amounts of biomass at sowing and effectively reach the upper asymptote when ready for harvest.

The SCRUM model assumes a fixed proportion of biomass is allocated to roots during growth. Root depth increases with thermal time and reaches a maximum at the end of the early reproductive phase (Fig. 1).

**Fig. 2:**
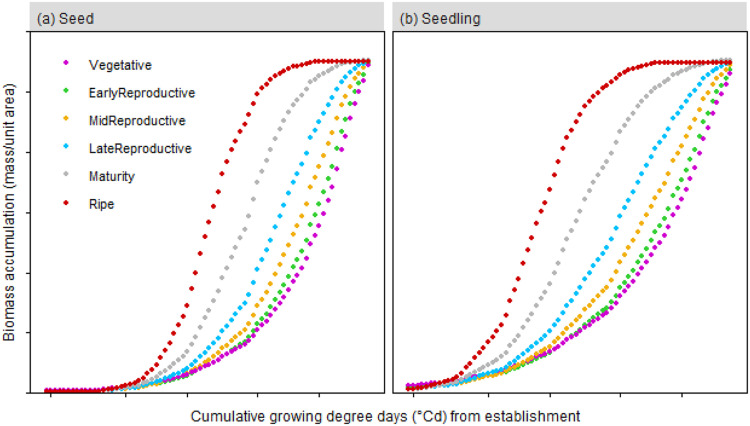
Biomass accumulation curve patterns for the Simple Crop Resource Uptake Model (SCRUM; based on the Agricultural Production Systems sIMulator (APSIM)) crops established and harvested on the same dates but established either by direct seeding(seed) or transplanting (seedling). Curves are shown for crops harvested at different developmental stages indicated in the legend.

Considering that crops are harvested at different growth stages depending on type or intended use, the model utilizes user-defined establishment and harvest stages to generate appropriate growth curves for biomass, canopy cover and root development. These curves are stretched between the specified establishment and harvest dates. An example of biomass accumulation for a crop sown from seed or transplanted and harvested at different stages is presented in Fig. 2. While this approach limits the model’s responsiveness to environmental variability and interannual changes, it ensures realistic growth and nitrogen uptake patterns when establishment and harvest dates are well defined.

### Model input parameters

SCRUM is not released with a set of predefined crop or cultivar parameterizations, unlike other APSIM crop modules. Instead, it has a user interface component that requires users to specify parameters for each crop in every simulation. These parameters are stored in a ‘SCRUM Crop Instance’ and define characteristics such as crop structure, resource acquisition, biomass growth and partitioning, tissue nitrogen content as well as temperature and water requirements (Table 2).

In addition to crop parameters, SCRUM requires the user to define crop management practices, which can vary widely across species. SCRUM uses a separate class called the ‘SCRUM Management Instance’ to handle management-related inputs. These are passed to the model each time a crop is sown, either from a manager script or another model that defines the crop rotation. This approach allows users to tailor practices to the specific crop or cultivar being simulated. The management parameters are grouped into four categories: crop specification, sowing and harvest schedules, crop yield setting, and residue handling (Table 3).

**Table 3:** Input parameters specified in a management instance of the Simple Crop Resource Uptake Model (SCRUM; based on the Agricultural Production Systems sIMulator (APSIM)).

**Define crop and basic management for SCRUM**	
Select crop to plant	SCRUM_Wheat
**Specify establishment and harvest conditions**	
Establishment date (dd-mmm or dd/mm/yyyy)	10/10/2020
Select stage of establishment	Seed|Emergence|Seedling
Planting depth (mm)	e.g. 20
Select how the crop cycle is defined	Harvest Date|Thermal-time requirement
Harvest date (dd-mmm or dd/mm/yyyy)	28/02/2021
Or Thermal time from establishment to harvest (°Cd)	e.g. 1200
Select stage at which the crop is harvested	Vegetative|Reproductive|Maturity|Ripe
**Specify potential yield of the crop (based on cultivar, sowing date and local conditions)**	
Potential crop yield (fresh weight; t/ha)	e.g. 8.0
**Specify proportions of field loss and residue management at harvest**	
Proportion of product retained in the field (0–1)	e.g. 0.05
Proportion of stover retained in the field (0–1)	e.g. 0.05
Proportion of residue incorporated at harvest (0–1)	e.g. 0.9
Depth to incorporate residue (mm)	e.g. 100

The flexibility to establish crops enables users to start crops from seed, emergence or transplant (seedling) stage (Table 3). This accommodates diverse practices. For example, crops typically sown directly may be transplanted as seedlings (raised in a nursery) if the growing season is short. When a crop is established by transplanting, the model bypasses the germination and emergence phases, starting the simulation from a seedling with an assigned proportion of biomass. In such cases, initial biomass and development progress are set to reflect the relative accumulated values at this stage (Table 1).

As previously mentioned, users can define crop duration (from establishment to harvest) using a calendar date or the thermal time (Table 3). SCRUM also supports varied harvest stages to reflect different crop end-uses such as vegetables, green feed forage, seed and grain. The proportion of unharvested biomass retained in the field varies by crop and end-use, and is managed through residue parameters, which also specify the residue composition (Table 3).

### Modelling biomass growth and resource uptake

**Fig. 3:**
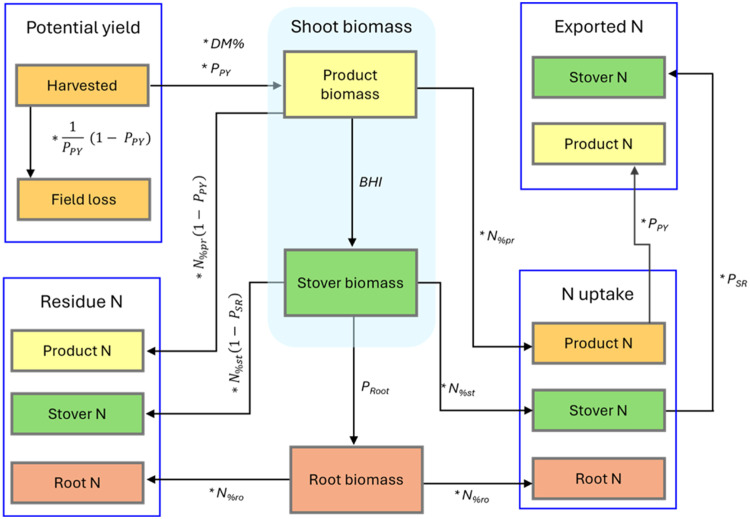
Schematic representation of nitrogen and biomass simulation in the Simple Crop Resource Uptake Model (SCRUM; based on the Agricultural Production Systems sIMulator (APSIM)). BHI = biomass harvest index, DM = dry matter, N = nitrogen, N_%pr_ = product N content, N_%ro_ = root N content, N_%st_ = stover N content, P_PY_ = harvested product proportion, P_SR_ = harvested stover proportion, P_Root_ = root proportion. * denotes multiply.

The model uses the species-specific expected (potential) yield provided by the user to estimate daily biomass production. This yield is converted into dry mass using the user-specified dry matter content (DM%) value (Fig. 3) and then to total above-ground biomass using a biomass harvest index (BHI), which is also specified by the user. A sigmodal function (Eq. (3)) is applied to determine the proportion of biomass produced each day. Daily biomass production is then partitioned to different plant parts (stover, product and root) based on crop-specific coefficients (harvest index and root proportion; Fig. 3), both of which are specified by the user.

To estimate daily nitrogen uptake, the dry mass of each organ is multiplied by its respective user-defined nitrogen content (Fig. 3), and the results are summed. Each organ has defined minimum, critical and maximum nitrogen contents. The minimum content represents the point below which growth is restricted. If nitrogen supply is insufficient to meet this minimum requirement for organs, the plant experiences stress, leading to reduced biomass production. The critical content represents the minimum amount required for maximum growth rate, while the maximum content is the amount at which nitrogen supply exceeds demand, resulting in luxury uptake.

For the root, both the critical and minimum nitrogen contents are set equal to the user-defined maximum content (Table 2) and remain constant throughout the lifespan of the crop. In contrast, the nitrogen content in stover and product organs varies during growth. It begins at a user-specified maximum value at seedling stage and declines to an organ-specific value at harvest (Table 2). The nitrogen content of the pre-seedling plant is assumed to be same as that of the seedling. The amount of nitrogen retained in the field is calculated as the sum of the nitrogen contained in the root biomass, the unharvested product and the unharvested stover (Fig. 3).

To model how nitrogen content of the biomass decreases during growth, a scaling factor is applied to the nitrogen content of stover and product tissues. This creates an exponential decay curve, which reflects the typical pattern of nitrogen dilution as the crop grows [17]. In each organ, the critical and minimum nitrogen contents are equal, remain constant throughout crop growth and are calculated as a fraction of the maximum nitrogen content at harvest.

Green canopy cover (m^2^ m^−2^) is required by SCRUM to estimate crop transpiration and soil evaporation and is modelled by a sigmoid function during canopy expansion and a linear function during canopy contraction, as described earlier.

Biomass production and cover expansion are sensitive to soil water availability. The effect of water stress is simulated by applying resource limitation factors ranging from zero (complete stress) to one (no stress) as described by Zheng, Chenu, Doherty and Chapman [18]. Water stress is triggered when the water supply in the root zone drops to 50% of the crop’s water demand.

### APSIM-STRUM simulation protocol

1. Set up the APSIM environment

**Fig. 4:**
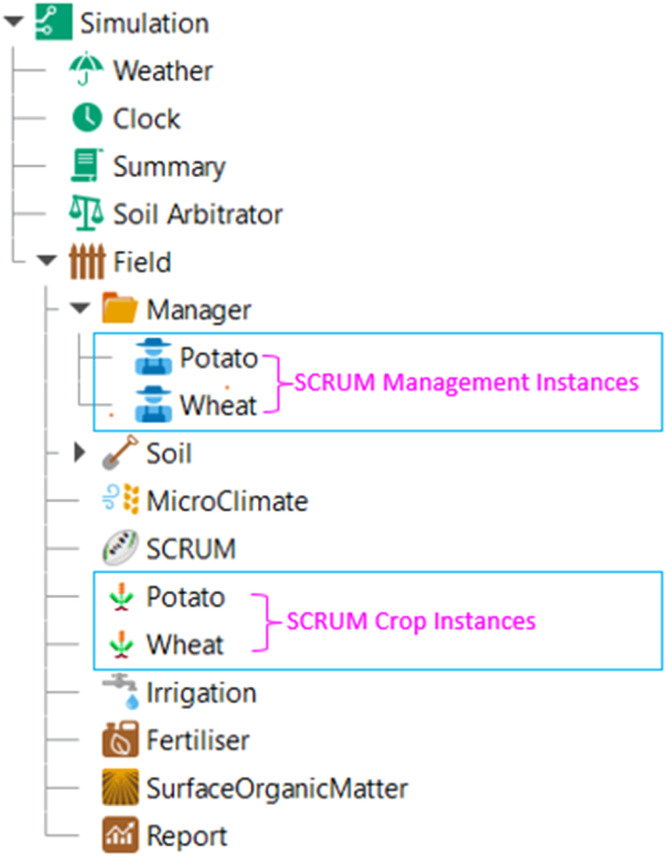
A snapshot of the components of the APSIM Field module showing SCRUM management and crop Instances.

A SCRUM crop model is added to the simulation in the same way as any other APSIM crop model. This is done by copying the SCRUM object from the APSIM crop folder in the toolbox and pasting it into the Field of the simulation (Fig. 4). SCRUM typically interacts with other modules within APSIM as shown in Fig. 4.

2. Generate SCRUM Crop Instance

A separate SCRUM Crop Instance is required for each crop included in the simulation. This can be done by duplicating an existing Crop Instance object and editing the parameters listed in Table 2 to match characteristics of the crop being simulated. Each crop in the simulation requires its own SCRUM Crop Instance (see example for potato and wheat crops in Fig. 4).

3. Create SCRUM Management Instance

A management instance includes the operations listed in Table 3. This can be created by copying an existing management object and editing it to match the management practices required for each crop in the simulation.

4. Organise outputs for reporting

Outputs are managed through the Report module. APSIM simulations run over the time period specified in the Clock module.

### Testing the sensibility and sensitivity of APSIM-SCRUM

To evaluate the performance and responsiveness of APSIM-SCRUM, a spring wheat crop was used as a case study. The aim was to assess whether the model produces outputs that are both agronomically sensible and responsive to environmental drivers. Specifically, the model’s sensitivity to variations in water and nitrogen supply was tested, alongside its ability to simulate changes in green cover and biomass accumulation under different establishment methods.

The Lincoln experimental field (43.63°S, 172.47°E, 12 m a.s.l.), operated by the New Zealand Institute for Bioeconomy Science Limited, was selected for test scenarios. This site is in the Canterbury region, where the majority of New Zealand wheat is grown [19], and features an on-site weather station [20]. The field sits on a deep (>1.6 m) Templeton silt loam soil, classified as *Udic Ustochrept* (U.S. Soil Taxonomy), which has a high water-holding capacity and has been used for numerous field crop experiments, including wheat [21].

In this test case, a modelled 8 t/ha SCRUM wheat grain crop (input parameters shown in Table 4) was established from seed on 20 September 2020 and harvested at the ripe stage on 28 February 2021.

**Table 4:** Crop input parameters used for the Simple Crop Resource Uptake Model (SCRUM; based on the Agricultural Production Systems sIMulator (APSIM)) wheat crop.

**Growth pattern and biomass partitioning parameters**	
Harvest index (HI; 0–1)	0.45
Product moisture content at harvest (g/g)	0.14
Root biomass proportion at maturity (0–1)	0.1
Root depth at maturity (mm)	1500
Crop height at maturity (mm)	1000
Maximum green cover (0–0.97)	0.97
Crop extinction coefficient (0–1)	0.7
Stage at which crop is typically harvested	Ripe
**Crop nitrogen (N) parameters**	
N content at seedling stage (g/g)	0.05
N content of product at harvest (g/g)	0.02
N content of stover at harvest (g/g)	0.01
N content of roots (g/g)	0.01
Potential N fixation by crop if legume (proportion; 0–1)	0
**Crop development temperature parameters**	
Crop base temperature (°C)	0
Crop optimum temperature (°C)	20
Crop maximum temperature (°C)	30
Thermal-time from sowing to emergence (°Cd)	150
**Crop water requirement parameters**	
Maximum canopy conductance (0.001–0.016)	0.008
Net radiation at 50% of maximum conductance (W/m^2^; 50–200)	150

To test the effect of resource supply, the modelled crop was cultivated under two irrigation conditions: rainfed and irrigated. Additionally, it was managed either with nitrogen fertilizer input (nitrogen-sufficient) or without fertilizer input (nitrogen-limited). Irrigation and nitrogen applications were scheduled automatically between 20 September and 15 December 2020. An irrigation event was triggered when moisture in the top 0.6 m of the soil profile dropped to ≤70% of the available water. Each event applied 15 mm of water. A minimum return period of four days and a refill threshold of 90% of the available water in the top 1 m of the soil profile were used in the model. Nitrogen fertilizer scheduling was based on regular soil tests. A total of 30 kg N/ha was applied when mineral nitrogen in the top 0.6 m of the soil profile dropped below 60 kg N/ha. A minimum interval of seven days was maintained between applications.

To assess crop growth response to different establishment methods, simulations require adjustments to the harvest date or thermal time requirements to complete the crop cycle. This is because crops starting from emergence or seedling stage begin their life cycle with a developmental advantage over those sown from seed. Harvest dates for emergence- and seedling-established crops can be estimated based on the number of days it takes a seed-sown crop to reach those respective stages. These days are then subtracted from the total crop cycle to shorten the time from establishment to harvest accordingly. In our example, the crop cycle was reduced by five days for emergence-stage establishment and by 11 days for seedling-stage establishment.

### Case study results and discussion

The implemented irrigation regime applied 263 mm to the nitrogen-sufficient treatments and 138 mm to nitrogen-limited treatments. Under these irrigated conditions, the average water uptake was 260 and 116 mm for nitrogen-sufficient and nitrogen-limited treatments, respectively. In comparison, rainfed treatments absorbed considerably less water, with uptakes of 151 mm and 91 mm estimated for nitrogen-sufficient and nitrogen-limited crops, respectively. During the growing season (September 2020–February 21), total rainfall was 207 mm, which was below the 30-year average of 255 mm (1993–2022). Every month of the season was drier than average, except November, which was wetter, and December, which received rainfall amounts comparable with the long-term average. For nitrogen-sufficient treatments, the model estimated nitrogen applications of 210 kg N/ha for irrigated and 150 kg N/ha for rainfed crops. Simulated nitrogen uptake values for the respective yields are consistent with results from a regionally matched spring wheat experiment [22]. Likewise, the modelled water uptake aligned with previously documented measurements for wheat crops grown in the same area [23].

**Fig. 5:**
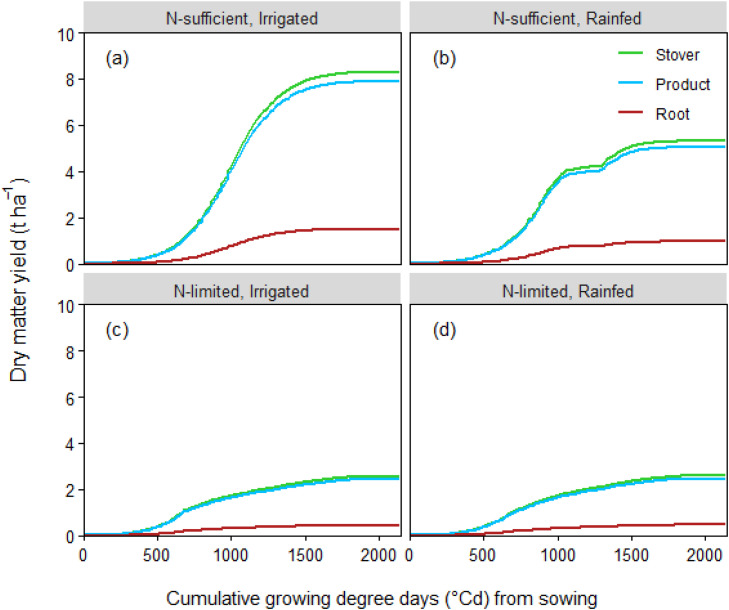
Effect of nitrogen and water supply on dry matter accumulation in different organs of a spring wheat crop as modelled by the Simple Crop Resource Uptake Model (SCRUM; based on the Agricultural Production Systems sIMulator (APSIM)).

Model outputs showed that APSIM-SCRUM successfully simulated the target yield of 8 t DM/ha under conditions of adequate water and nitrogen supply (Fig. 5a). When nitrogen, water or both were limited, the model predicted varying degrees of dry matter reduction (Fig. 5c–d). The greatest reduction occurred under simultaneous limitation of both water and nitrogen, highlighting the compounded impact of stress on plant growth. This response aligns with the model’s application of a water stress correction factor, which reduces growth when water supply is insufficient. The simplified approach of applying water stress factor to limit plant growth, thereby indirectly reducing nitrogen uptake, mirrors research findings that show nitrogen uptake is strongly influenced by soil moisture availability [24]. These results indicate that APSIM-SCRUM has the potential to simulate irrigation-nitrogen interactions in a realistic and agronomically meaningful way, supporting its use in exploratory analyses of resource-limited cropping systems.

**Fig. 6:**
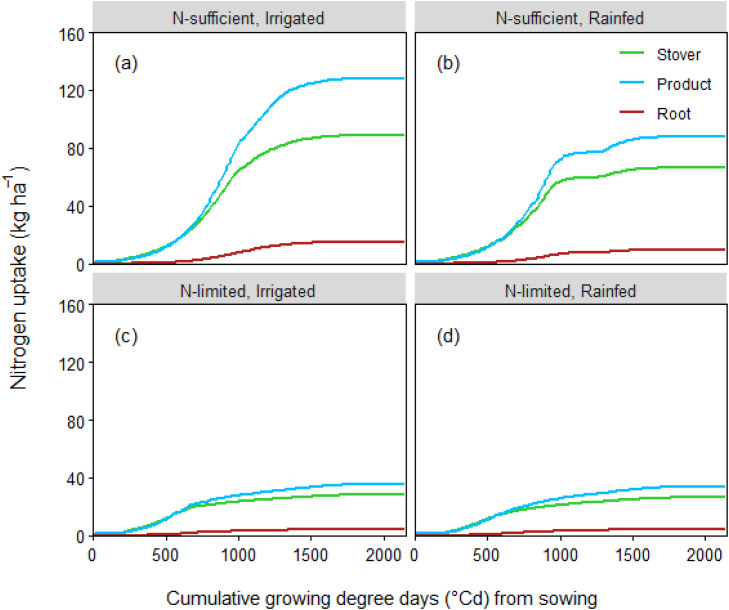
Nitrogen and water supply effect on nitrogen taken up by different organs of a spring wheat crop as modelled by the Simple Crop Resource Uptake Model (SCRUM; based on the Agricultural Production Systems sIMulator (APSIM)).

Nitrogen uptake by various plant organs mirrored the patterns of dry matter accumulation, as shown in Fig. 5, Fig. 6. This outcome aligns with the expectations, given that SCRUM models nitrogen uptake based on estimated daily biomass and organ-specific tissue nitrogen content coefficients, while also accounting for soil nitrogen availability. The lower nitrogen uptake predicted under ‘nitrogen-sufficient, rainfed’ conditions compared with ‘nitrogen-sufficient, irrigated’ scenarios reflects the physiological limitation of reduced transpiration-driven mass flow of nitrogen ions, a process that is constrained when water availability is reduced [25].

**Fig. 7:**
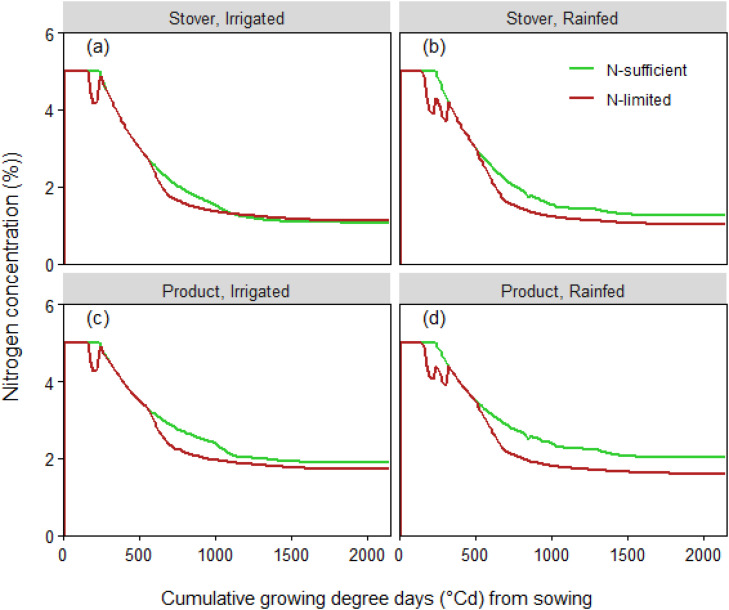
Nitrogen and water supply effect on tissue nitrogen content of spring wheat organs as modelled by the Simple Crop Resource Uptake Model (SCRUM; based on the Agricultural Production Systems sIMulator (APSIM)).

The graphs in Fig. 7 demonstrate a progressive decline in tissue nitrogen content in both stover and product organs as biomass accumulates, consistent with the concept of nitrogen dilution. This indicates that the simple approach of applying a scaling factor to the nitrogen content between the seedling stage and the harvest stage successfully generated a pattern that mirrors tissue nitrogen dynamics during crop growth. Differences in tissue nitrogen content between nitrogen treatments became evident after crops accumulated 500–600 °Cd from sowing, coinciding with the onset of rapid growth (Fig. 5). These differences persisted throughout crop development except in stover under irrigated conditions, where the gap diminished. This probably occurred because the nitrogen-limited treatments produced proportionally less biomass relative to available soil nitrogen, reducing the dilution effect.

**Fig. 8:**
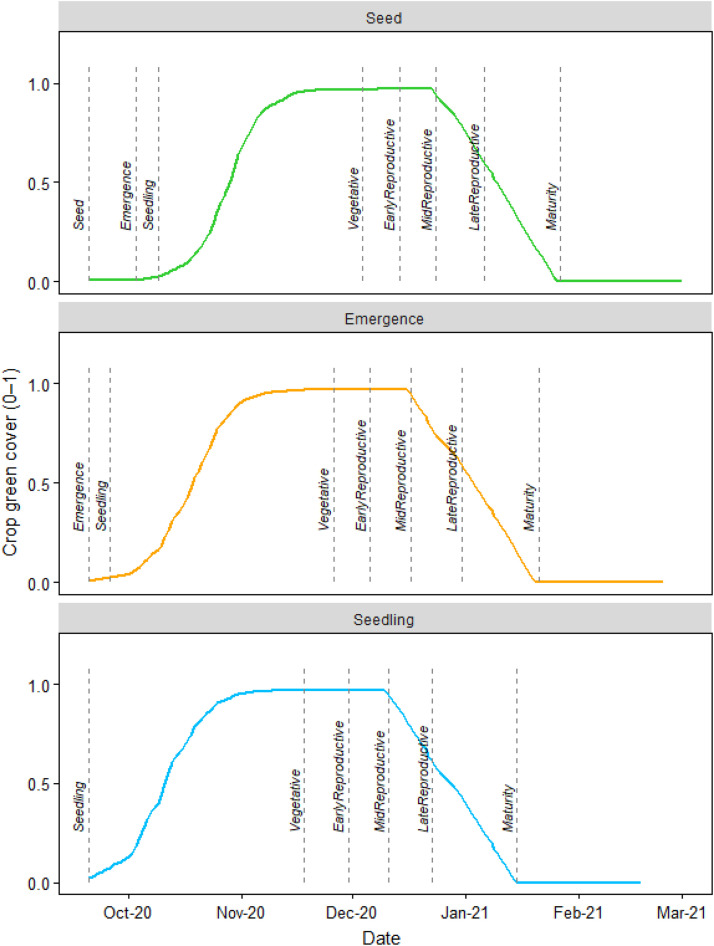
Changes in ground green cover (m^2^ m-^2^) for a generic crop started at the seed, emergence, seedling stage and harvested at varied developmental stages (vegetative, early reproductive, mid-reproductive, late-reproductive, maturity, ripe) as predicted by the Simple Crop Resource Uptake Model (SCRUM; based on the Agricultural Production Systems sIMulator (APSIM)).

**Fig. 9:**
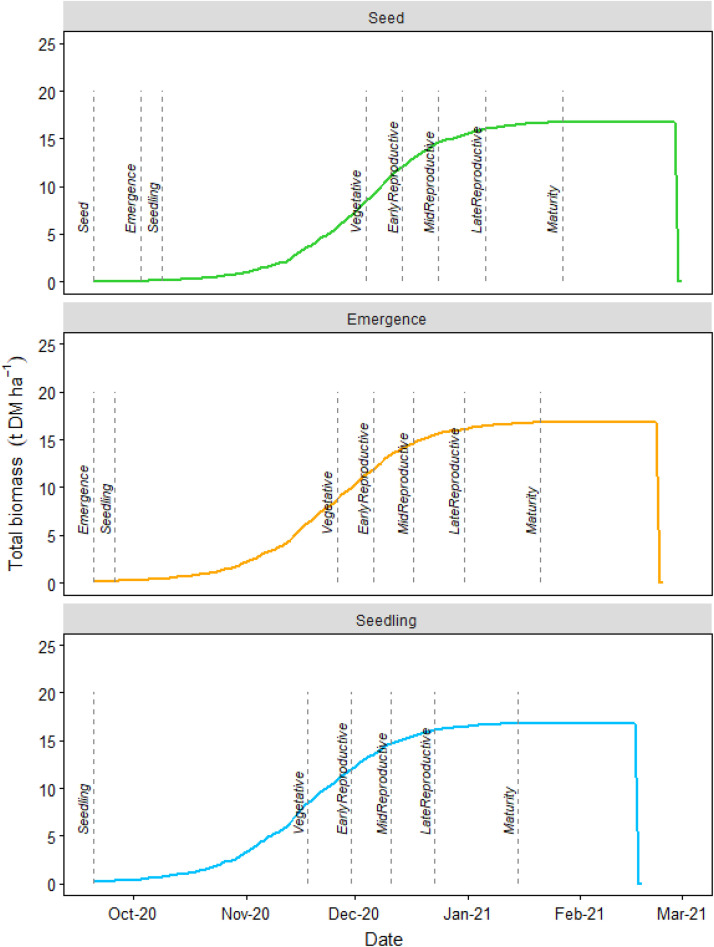
Changes in total biomass for a generic crop started at the seed, emergence, seedling stage and harvested at varied developmental stages (vegetative, early reproductive, mid-reproductive, late-reproductive, maturity, ripe) as predicted by the Simple Crop Resource Uptake Model (SCRUM; based on the Agricultural Production Systems sIMulator (APSIM)).

The development of the crop green cover shows differences between establishment methods (Fig. 8). As expected, crops established from seedlings developed faster and achieved maximum cover earlier than those established from emergence, which were in turn earlier than seed-sown crops. Likewise, biomass accumulation was slower when crops were established from seed, intermediate for establishment from emergence, and fastest when transplanted (Fig. 9). The stage at which the crop is harvested determines the amount of biomass produced, with the maximum achieved at maturity. Graphs in Fig. 9 are based on crops being sufficiently supplied with nitrogen and water. Under these conditions, the amount of biomass produced would be related to the stage of harvest, i.e. the earlier the stage, the lower the amount of biomass (Fig. 9).

## Method validation

### Data sources

The performance of APSIM-SCRUM was assessed using data from maize and vegetable experiments conducted at different New Zealand locations. The maize experiment was carried under rain-out shelter conditions at Lincoln, Canterbury (43°37′26.55“S, 172°28′4.46″E) during the 2012–23 season. The trial evaluated maize growth and nitrogen uptake responses to two water treatments (nil (non-irrigated) and full irrigation) and three nitrogen treatments (fertiliser input of 0, 75 and 250 kg N/ha). Details of the are provided in Teixeira et al. [26].

The vegetable trials consisted of two crop rotation experiments carried out at two sites. The first rotation (wheat–broccoli–onion–ryegrass) was established at Lincoln in Canterbury (43°37′32.51“S, 172°28′1.10″E) while the second rotation (pak choi–lettuce–pea) was established at Hastings in Hawke’s Bay (43°37′32.37“S, 172°28′6.64”E). Crops were subjected two irrigation regimes (Irrigation 1 and Irrigation 2) and four fertiliser nitrogen treatments (N1, N2, N3 and N4) as shown in Table 5. Details of these crop rotation experiments are described by Khaembah et al. [27] and Searle et al. [28].

**Table 5:** Irrigation water and fertiliser nitrogen (N) applied to crops in rotation at Lincoln (Canterbury) and Hastings (Hawke’s Bay), New Zealand.

Location	**Crop in rotation**	**Applied irrigation (mm)**		**Applied fertiliser (kg N/ha)**			
		**Irrigation 1**	**Irrigation 2**	**N1**	**N2**	**N3**	**N4**
Lincoln	Wheat	330	375	150	150	150	150
	Broccoli	135	135	0	30	60	120
	Onion	155	205	0	60	120	240
	Ryegrass	165	215	29	89	149	269
Hawke’s Bay	Pak-choi	35	35	0	19	38	75
	Lettuce	15	15	0	30	60	120
	Peas	75	75	0	0	0	0

### Analysis of model performance

The prediction accuracy of the model was evaluated using four indices: the coefficient of determination (R^2^), the root mean squared error (RMSE), the RMSE-observations standard deviation ratio (RSR) and the Nash – Sutcliffe efficiency (NSE), all of which are built into the APSIM test suite. A normalised RMSE (NRMSE), which measures the relative magnitude of error compared to the average value of the observed data, was also included. The RSR varies from an optimal value of 0 upwards and is classified as follows: 0 < RSR ≤ 0.5 (very good), 0.5 < RSR ≤ 0.6 (good), 0.6 < RSR ≤ 0.7 (satisfactory), RSR > 0.7 (unsatisfactory) are considered to describe very good, good, satisfactory and unsatisfactory model prediction accuracy, respectively. The NSE ranges from –∞ to 1, with 1 indicating a perfect fit. Performance thresholds are 0.75 < NSE ≤ 1 (very good), 0.65 < NSE ≤ 0.75 (good), 0.5 < NSE ≤ 0.65 (satisfactory), NSE ≤ 0.5 (unsatisfactory).

**Fig. 10:**
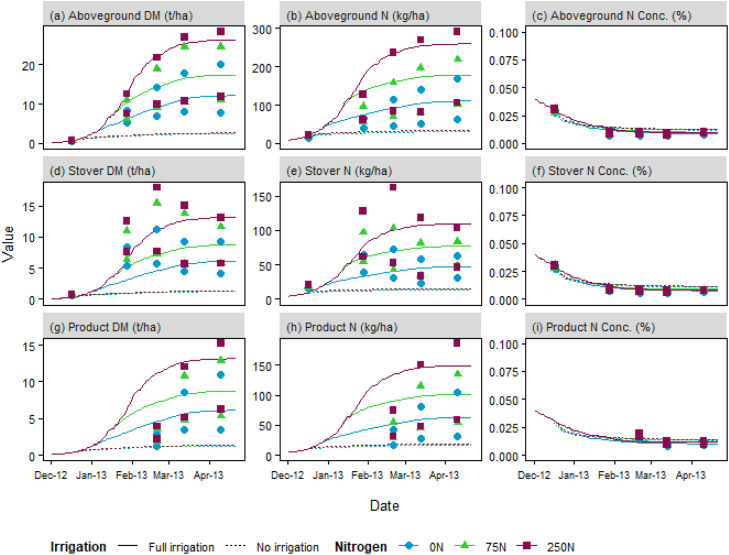
Measured (symbols) and APSIM-SCRUM–predicted (lines) changes in dry matter (DM) content, nitrogen (N) content and tissue N concentration in different organs of maize grown under two irrigation regimes and three N fertiliser treatments.

**Fig. 11:**
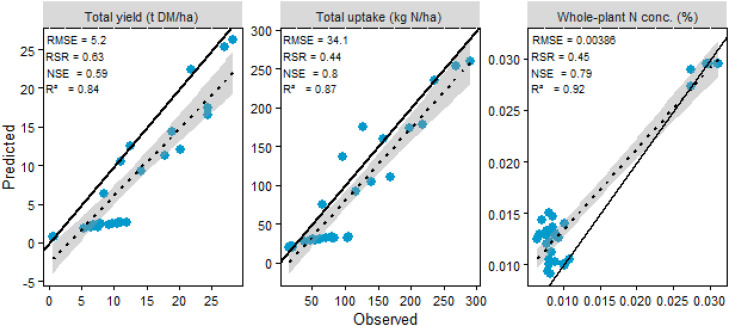
The APSIM-SCRUM–predicted versus measured values of aboveground dry matter (DM), nitrogen (N) content and tissue N concentration of maize grown under two irrigation regimes and three N fertiliser treatments.

The data in Fig. 10 show that the model captured the overall patterns of maize growth, nitrogen uptake and tissue nitrogen concentration, especially under adequate water supply. SCRUM uses yield as an input, so the irrigated 250 kg N/ha treatment yield was used to define the model’s reference yield, with water and nitrogen stress functions reducing yield under limiting conditions. The model reduced yield when resources were restricted although it appeared excessively sensitive, leading to under-estimation of yield in the nil nitrogen and irrigation treatments. Overall, prediction accuracy ranged from satisfactory to very good as indicated by the Observed versus estimated scatter plots and performance indicators shown in Fig. 11. On a whole-plant basis, NRMSE values indicated that the simulated data accounted for the majority of the variation in the observed data; 54, 66 and 69% for yield, nitrogen uptake and tissue nitrogen concentration, respectively (Fig. 11).

**Fig. 12:**
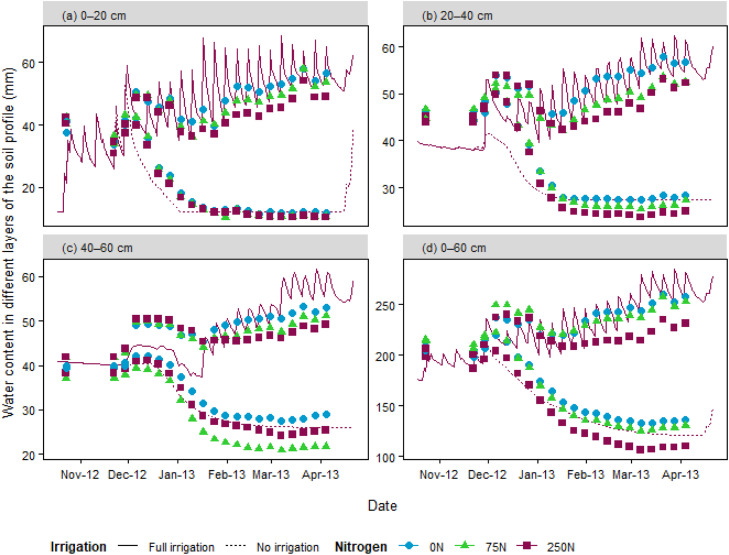
Measured and APSIM-SCRUM–predicted soil water content in different soil layers for maize grown under two irrigation regimes and three N fertiliser treatments.

Comparison of soil water content across the different soi layers shows that the model captured the separation between irrigated and non-irrigated treatments well (Fig. 12). Although differences among the nitrogen treatments were indistinguishable, the overall seasonal pattern in soil water content was well represented.

**Fig. 13:**
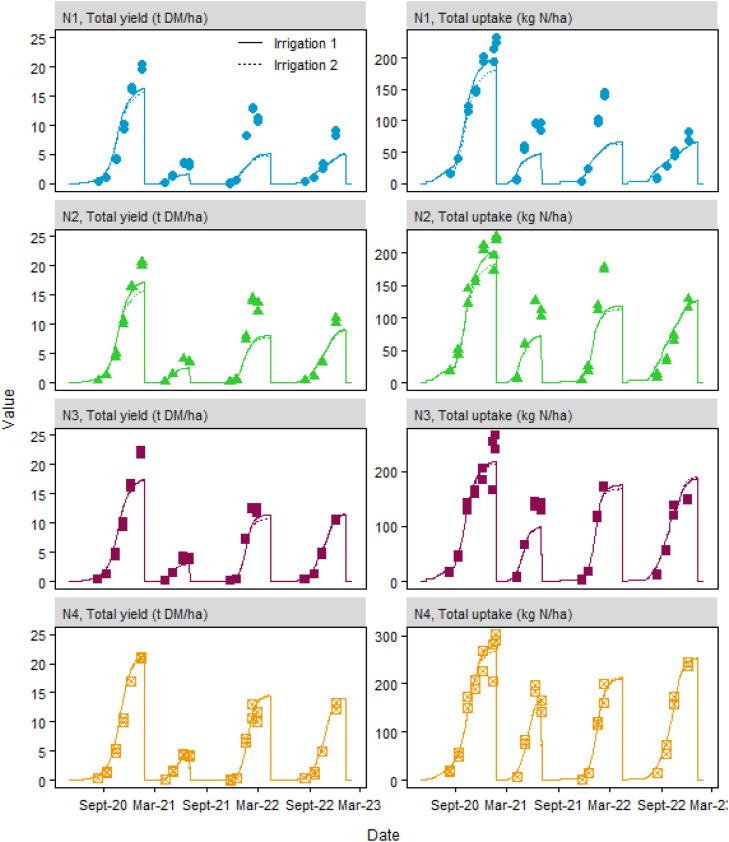
Measured (symbols) and APSIM-SCRUM–predicted (lines) changes in dry matter (DM) content and nitrogen (N) content for crops grown in rotations under two irrigation levels and four fertiliser N treatments. Crops in rotation (wheat–broccoli–onion–ryegrass) were established at Lincoln, Canterbury, New Zealand.

Comparison of data based on the crop rotation experiments also showed that APSIM-STRUM responded to resource limitations by reducing yield at the Lincoln site (Fig. 13). Yield was notably under-estimated for some crops particularly under the N1 nitrogen treatments. However, the seasonal patterns of nitrogen accumulation, including the effects of resource limitations, were well captured by the model. There were marginal differences between irrigation managements at Lincoln, where irrigation input varied substantially between irrigation treatments. Where irrigation inputs were similar between irrigation treatments (e.g. broccoli; Table 5) or when seasonal rainfall was above average (e.g. ryegrass; Table 5), no differences were observed (Fig. 13).

**Fig. 14:**
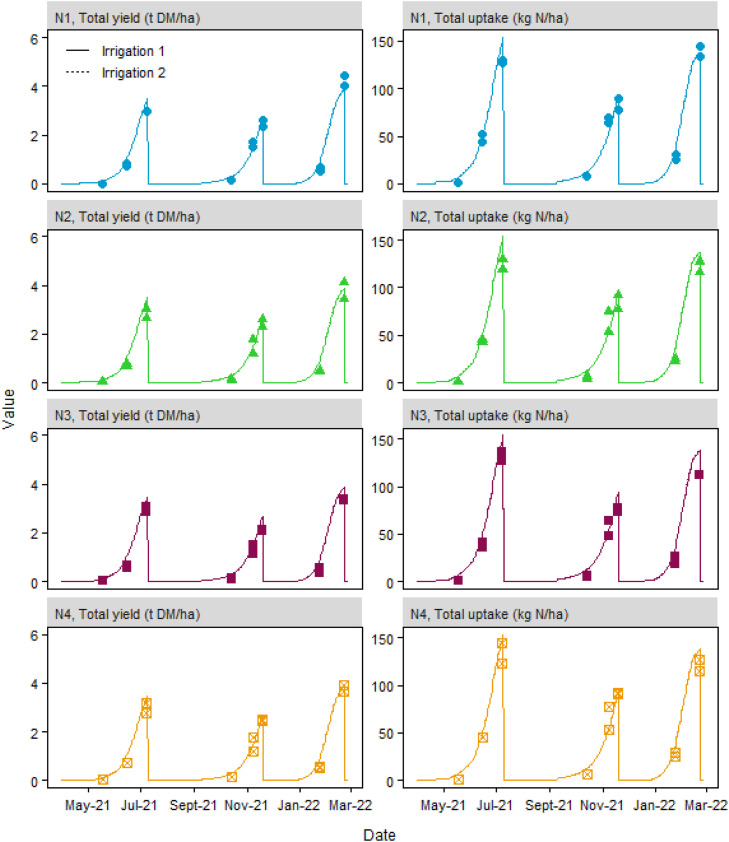
Measured (symbols) and APSIM-SCRUM–predicted (lines) changes in dry matter (DM) content and nitrogen (N) content for crops grown in rotations under two irrigation levels and four fertiliser N treatments. Crops in rotation (pak choi–lettuce–pea) were established at Hastings, Hawke’s Bay, New Zealand.

At Hawke’s Bay, the model accurately reproduced the seasonal trends in yield and nitrogen uptake (Fig. 14). All crops received the same amount of irrigation water (Table 5), which explains the lack of difference between irrigation treatments.

**Fig. 15:**
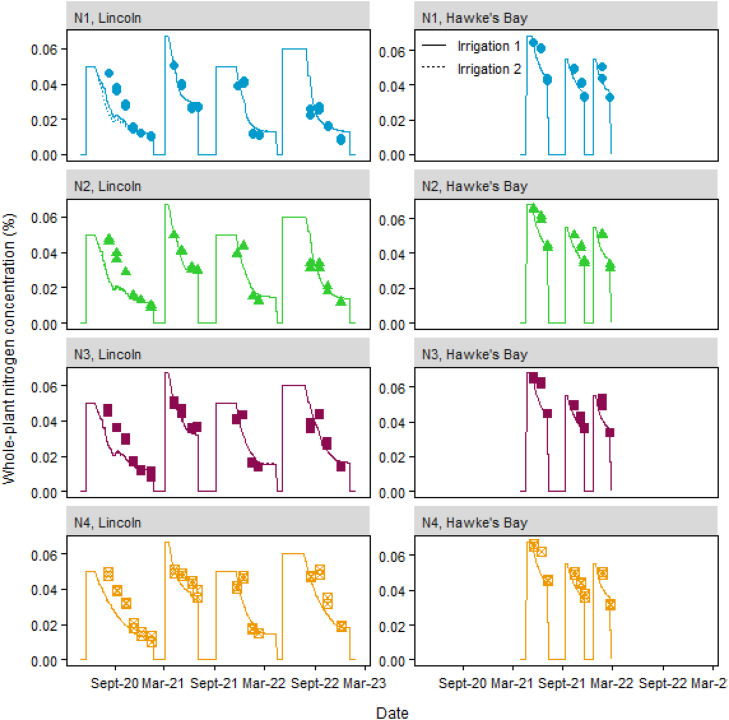
Measured (symbols) and APSIM-SCRUM–predicted (lines) changes in aboveground biomass nitrogen (N) concentration for crops grown in rotations under two irrigation levels and four fertiliser N treatments. The Lincoln crops in rotation (wheat–broccoli–onion–ryegrass) were established at Lincoln, Canterbury and the Hawke’s Bay crops in rotation (pak choi–lettuce–pea) were established at Hastings, Hawke’s Bay, New Zealand.

**Fig. 16:**
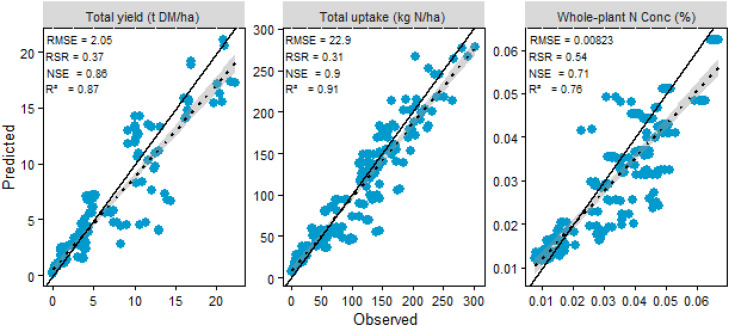
The APSIM-SCRUM–predicted versus measured values of aboveground dry matter (DM), nitrogen (N) content and tissue N concentration for crops grown in rotations under two irrigation levels and four fertiliser N treatments. The Lincoln crops in rotation (wheat–broccoli–onion–ryegrass) were established at Lincoln, Canterbury and the Hawke’s Bay crops in rotation (pak choi–lettuce–pea) were established at Hastings, Hawke’s Bay, New Zealand.

The graphical evaluation and performance metrics for dry matter, nitrogen content and tissue nitrogen concentration in the aboveground biomass (Fig. 13, Fig. 14, Fig. 15, Fig. 16) show that model performance ranged from satisfactory to very good (R^2^ = 0.76–0.91, RSR = 0.31–0.54, NSE = 0.71–0.90) across rotational crops. Overall, the simulated data explained 55–77% variation in the measured values.

**Fig. 17:**
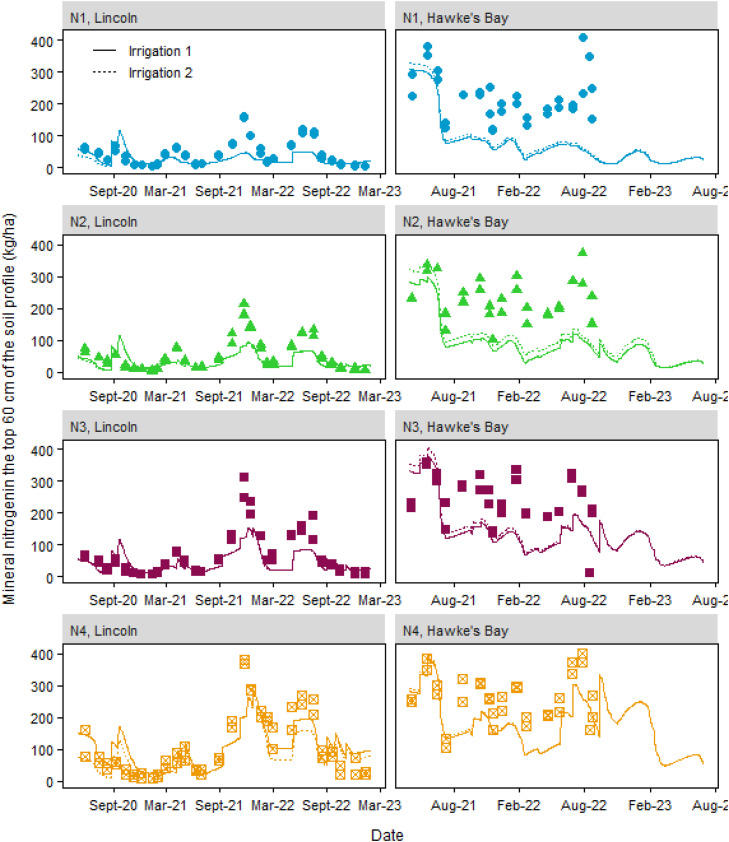
Measured and APSIM-SCRUM–predicted soil nitrogen content (0–60 cm) for crops grown in rotations under two irrigation levels and four fertiliser N treatments. The Lincoln crops in rotation (wheat–broccoli–onion–ryegrass) were established at Lincoln, Canterbury and the Hawke’s Bay crops in rotation (pak choi–lettuce–pea) were established at Hastings, Hawke’s Bay, New Zealand.

The temporal patterns of soil mineral nitrogen in the top 60 cm (Fig. 17) indicate that predicted values generally followed the same trend as the measurements at Lincoln (R^2^ = 0.70, RSR = 0.67, NSE = 0.55). Model fit was weaker at Hawke’s Bay (R^2^ = 0.23, RSR = 1.64, NSE = –1.72), where soil mineral nitrogen was mostly under-estimated. While spatial variation in soil nitrogen measurements is a common contributor to the discrepancies. The poorer performance at Hawke’s Bay may also reflect the presence of mineral water in the shallow groundwater. Upward fluxes of nitrogen from the water table are not represented in the model, and this unaccounted for input may have contributed to the under-estimation of soil mineral nitrogen.

**Fig. 18:**
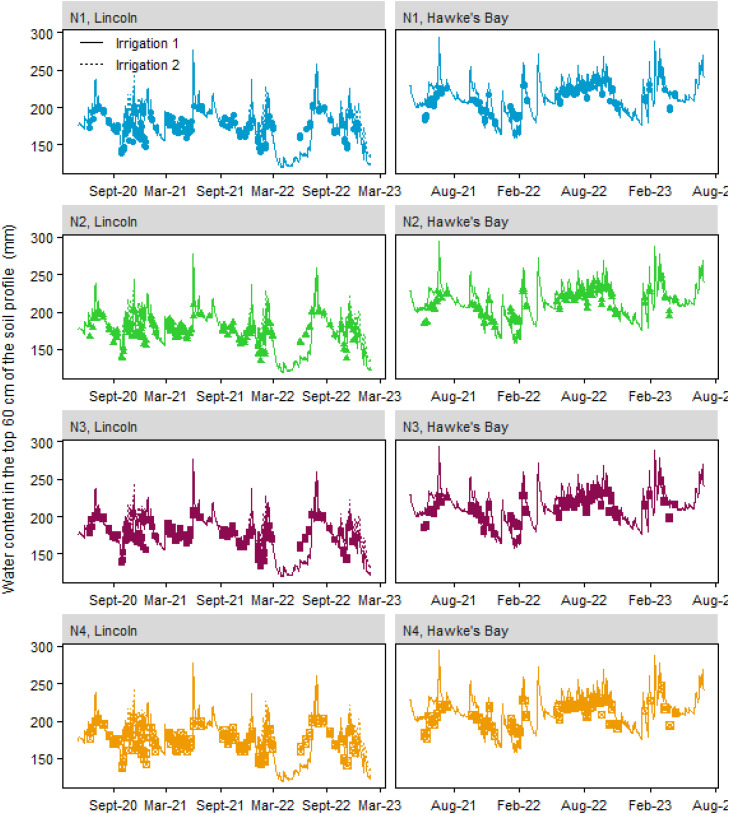
Measured and APSIM-SCRUM–predicted soil water content (0–60 cm) for crops grown in rotations under two irrigation levels and four fertiliser N treatments. The Lincoln crops in rotation (wheat–broccoli–onion–ryegrass) were established at Lincoln, Canterbury and the Hawke’s Bay crops in rotation (pak choi–lettuce–pea) were established at Hastings, Hawke’s Bay, New Zealand.

Temporal changes in soil water content (Fig. 18) indicate that APSIM-SCRUM captured the overall trends and produced good estimates of seasonal soil moisture in the top 60 cm of profile at both crop rotation sites. Overall model bias was small (approximately 4%) and the simulated data explained 92% of the variation in the data (NRMSE = 8.0). Other performance metrics (R^2^ = 0.77, RSR = 0.63, NSE = 0.61) supported a satisfactory model fit (Fig. 18).

## Discussion

This study presents the development of SCRUM in APSIM, a simplified and adaptable model designed to predict water and nitrogen balance in annual cropping systems. The generic and simple nature of SCRUM allows for the easy integration of new crops or cultivars based on expert knowledge, making it particularly valuable for simulating diverse and dynamic crop rotations. This adaptability is a critical requirement in arable and vegetable crop systems, where species and cultivar composition frequently change and may include emerging or under-researched crops.

As an initial verification step, we confirmed that under non-limiting water and nitrogen, the model reproduced the user-specified target yield, indicating that APSIM-SCRUM behaved consistently with its input assumptions. With the baseline confirmed, model performance was evaluated under resource-limited scenarios. When subjected to water stress, nitrogen stress and combined limitations, APSIM-SCRUM generated the expected yield penalties, indicating realistic sensitivity to individual and interacting limitations. These responses align well-established physiological principles, particularly the coupling of transpiration with nutrient uptake, and together demonstrate the model’s ability to simulate interacting stress effects across variable environmental conditions.

Evaluation of the model using measured data from two New Zealand regions showed that APSIM-SCRUM generally reproduced the observed changes in crop nitrogen accumulation, tissue nitrogen concentration, soil moisture and soil mineral, as demonstrated by the model performance metrics. A few simulations were less accurate, partly because APSIM does not currently include a function to represent the effects of a shallow water table. Overall, APSIM-SCRUM captured the key trends and exhibited sensitivity to water and nitrogen supply, indicating potential as a tool for analysing nitrogen and water management practices of across a range of conditions.

Beyond the verification assessments, SCRUM’s design offers notable advantages for advancing sustainable crop production and climate change research. As growing conditions become increasingly variable because of changing weather patterns and altered seasonal cycles, the ability to rapidly adapt simulations to new scenarios is critical. SCRUM supports this adaptability by enabling users to easily modify crop and management parameter inputs such as planting dates, crop types and expected yield across diverse environments. This flexibility enables researchers to evaluate the performance of standard and novel crops under varying growing conditions, and to explore a wide range of adaptation strategies. In turn, the ability to assess crop performance under projected climate futures is crucial for developing resilient and adaptable farming systems.

Unlike other crop models, SCRUM requires users to specify potential yield and harvest dates as inputs rather than predicting them as outputs. This design feature simplifies crop representation and enhances generality, but it also constrains the model’s applicability in scenarios where these variables may shift substantially. Nevertheless, it is assumed that in many practical contexts users can provide reasonable estimates, drawing on agronomic expertise, historical records or published scenario-based projections, which helps keep the model simple while maintaining broad applicability.

Finally, SCRUM supports multiple crop instance parameterizations within a single simulation and enables their repeated use across long-term sequences. This capability makes it possible to represent complex, extended crop rotations and to integrate with other APSIM plant models, greatly broadening the range rotation scenarios that can be explored.

### Limitations

SCRUM is designed as a simplified framework, which limits its general applicability compared with full crop models such as APSIM-Wheat and APSIM-Maize. Users should therefore exercise caution when adjusting parameters for specific applications. Inputs should be appropriate based on the crop type, location and prevailing growing conditions. For example, potential yield for a given crop may vary significantly between different regions because of climate, soil type and management practices, and such differences should be reflected in the parameter choices. Sensibility testing or basic validation to confirm that outputs align with expected crop performance is strongly recommended prior to applying the model.

There are structural assumptions that constrain the model’s ability to represent species-specific dynamics and resource partitioning among organs. These include:

• Potential yield and harvest dates (or thermal time) are model inputs rather than outputs, limiting its applicability when these variables vary significantly across scenarios.
• Uniform growth dynamics is assumed—all crops follow the same sigmoidal growth curve irrespective of species.
• The proportion of thermal time allocated to each phenological phase is the same irrespective of the crop species.
• Nitrogen content and biomass allocation to roots are held constant, assuming no adjustments during crop development.
• SCRUM allocates biomass to all organs starting at crop emergence, whereas in reality, product development in crops such as grains typically begins during the reproductive phase.

The simplifications support broader applicability and ease of use but may limit accuracy in scenarios requiring detailed physiological representation.

## Supplementary material *and/or* additional information [Optional]

None.

## Related research article

None.

## Ethics statements

None.

## CRediT author statement

Edith N. Khaembah: Investigation (model testing); Methodology (model refinements); Writing – Original Draft. Rogerio Cichota: Methodology (model refinements). Megan Gee: Writing – Review & Editing (edited and formatted references in the final manuscript). Hamish Brown: Software (model development); Writing – Review & Editing.

## Declaration of AI-assisted technology

During the preparation of this manuscript, Edith Khaembah used Microsoft CoPilot to assist with refining text and improving clarity. All content generated with AI assistance was reviewed and edited by the author, who takes full responsibility for the final version of this manuscript.

## Declaration of competing interest

The authors declare that their organisation (New Zealand Institute for Bioeconomy Science Limited) is a member of the APSIM Initiative, which manages the development and governance of the APSIM model. This membership did not influence the conduct or outcomes of this study.

## Data Availability

Data will be made available on request.
